# Optimizing a desirable fare structure for a bus-subway corridor

**DOI:** 10.1371/journal.pone.0184815

**Published:** 2017-10-05

**Authors:** Bing-Zheng Liu, Ying-En Ge, Kai Cao, Xi Jiang, Lingyun Meng, Ding Liu, Yunfeng Gao

**Affiliations:** 1 School of Transportation and Logistics, Faculty of Infrastructure Engineering, Dalian University of Technology; Dalian, Liaoning Province, China; 2 College of Transport & Communications, Shanghai Maritime University; Shanghai, China; 3 School of Transportation and Vehicle Engineering, Shandong University of Technology; Zibo, Shandong Province, China; 4 State Key Laboratory of Rail Traffic Control and Safety, Beijing Jiaotong University, HaiDian District, Beijing, China; Beihang University, CHINA

## Abstract

This paper aims to optimize a desirable fare structure for the public transit service along a bus-subway corridor with the consideration of those factors related to equity in trip, including travel distance and comfort level. The travel distance factor is represented by the distance-based fare strategy, which is an existing differential strategy. The comfort level one is considered in the area-based fare strategy which is a new differential strategy defined in this paper. Both factors are referred to by the combined fare strategy which is composed of distance-based and area-based fare strategies. The flat fare strategy is applied to determine a reference level of social welfare and obtain the general passenger flow along transit lines, which is used to divide areas or zones along the corridor. This problem is formulated as a bi-level program, of which the upper level maximizes the social welfare and the lower level capturing traveler choice behavior is a variable-demand stochastic user equilibrium assignment model. A genetic algorithm is applied to solve the bi-level program while the method of successive averages is adopted to solve the lower-level model. A series of numerical experiments are carried out to illustrate the performance of the models and solution methods. Numerical results indicate that all three differential fare strategies play a better role in enhancing the social welfare than the flat fare strategy and that the fare structure under the combined fare strategy generates the highest social welfare and the largest resulting passenger demand, which implies that the more equity factors a differential fare strategy involves the more desirable fare structure the strategy has.

## 1 Introduction

Public transit composed of bus, subway, taxi, etc. is of great importance to meet the need for urban individual mobility and, as a mean of travel demand management, to mitigate traffic congestion [1–3]. The comprehensive effectiveness of such an integrated transit system can only be achieved with the help of consolidated transit fare structure. As a flexible instrument, public transit service fare influences passenger behavior directly, e.g. whether to travel, where to travel, when to travel and how to travel, etc. To find a desirable fare structure, it is necessary to identify the most suitable fare strategy. This paper considers a public transit corridor served by bus and subway lines, which is termed a bus-subway corridor in the of the paper.

The optimization of transit fare structure has been widely investigated in the literature on transportation economics [4–5] and network equilibrium analysis [6–7]. The relevant studies can be traced back to the 1970s, e.g. Nash [8] and Glaister and Collings [9], which proposed treating the design of a fare structure as an optimization problem. They respectively applied an elastic-demand function and a linear demand function to capture the influence of travel costs on passenger behavior without regard to externalities (e.g. congestion). Spiess and Florian [10] proposed a new assignment model for transit networks as an alternative way to find the optimal strategy for public transit service plan. De Borger and Wouters [11] investigated transport externalities, optimal pricing and supply decisions in urban transportation systems for Belgium by means of a simulation analysis technique. Li et al. [12] considered the optimal transit fare structure under different market regimes with uncertainty in a network. Tirachini et al. [13] concerned multimodal pricing and optimal design of urban public transport with a focus on the interplay between traffic congestion and bus crowding. Kaddoura et al. [14] found a range of values for the optimal fare and headway by means of an agent-based approach considering the randomness in user behavior. de Palma et al. [15] derived the optimal time table and the optimal pricing considering crowding in public transport and its implications for pricing, capacity design and optimal scheduling.

With the diversification of urban individual travel, the issue of coordinated pricing for different transit modes, or taxi and even private cars in is a focus in the literature on public transit service provision. Li et al. [16] optimized a bus-rail transit system with feeder bus services under different market regimes. Lu et al. [17] enhanced the insights into pricing mechanism for subway and parking and corresponding mode choice behavior on the corridor with elastic demand.

There are two basic categories of existing fare strategies: flat one which requires all passengers to pay the same fare and differentiated one which allows passengers to pay a fare varying in a way to respond to several factors in their trips. These and other fare strategies are listed below [18–19].

1. *Flat fare*: passengers are charged the same fare.
2. *Distance-based or zonal fare*: A fare determined by the distance or amount of zones a trip covers.
3. *Time-based fare*: A fare that depends on when to start and how long a trip lasts.
4. *Quality-based fare*: A fare related to which service a passenger receives, e.g. express, short-turn or local services.
5. *Cost-based fare*: A fare based on operating cost, e.g. air-conditioning cost or staff wages.
6. *Route-based fare*: A fare associated with which zones a bus goes through, such as CBD, residential zones, work places, or tourist places.
7. *Patron-based fare*: A fare that depends on types of passengers, such as students, senior citizens, or disabled passengers.
8. *Market- or consumer-based fare*: A fare that depends on the frequency of use and willingness to pay, such as passes and discounted tickets.

All of these have been discussed in the literature. It is widely acknowledged that the flat fare structure is the simplest and most convenient one [18] while the differential fare structure is not only more competent in addressing social equity concern but also can gain a greater increase in the operator’s revenue [19–23]. Chien and Tsai [19] proposed an optimization model developed for optimizing operational headway and differential time- and zone-based fare structures, taking service capacity constraints into account. Ling [20] evaluated the effects of flat and differential fare structures on passenger travel demand, revenues, passenger-km, and consumer surplus. Borndörfer et al. [21] discussed the effects of distance-based and single/monthly fare structures on passengers’ travel behavior. Tsai et al. [22] also illustrated such significant impacts of distance-based fare on passengers’ travel behavior in an intercity transit system. Zhang et al. [23] analyzed the positive impacts of different fare structures in different kinds of adverse weather conditions. Ge et al. [2–3] proposed a visualized fare table for the design of limited-stop bus services along a public transit corridor. Tang et al. [24] proposed an optimization approach to design of a transit service system under elastic demand. In this approach, the objective is to maximize the total social welfare, by providing a profitable fare structure and tailoring operational strategies to passenger demand. The demand function is dependent not only on the attributes of these strategies, in-vehicle crowding but also on the effects of the fare on demand variation, where the fare is either a flat or a differential fare structure. The results indicate that an optimal combination of the operational strategies integrated with a differential fare structure results in the highest potential for increasing the total social welfare. To the best of our knowledge, neither of the existing studies focused on the integrated fare structure in such a bus-subway corridor nor did they identify the more suitable differential fare structure for a corridor. This paper aims to propose fare structures combining flat fare strategy and more than one differential fare strategies considering the request of social equity and investigate effects of different fare strategies on optimal fare structure and passenger behavior, and then help decision makers determine the optimal fare structure.

Two criteria are applied to measure and determine the most desirable or optimal fare structure within several fare strategies proposed in this research. The objective of this problem is to pursue the maximum social welfare of the public transit service and the constraints include travel distance and comfort level in passengers' trip, which are related to the social equity issue. The study in this paper can be summarized in the following two steps:

**Step 1**. The flat fare is applied to obtain an optimal social welfare reference and the general distribution of passengers along a corridor under investigation. The whole transit corridor can be divided into comfortable (or crowding) area and uncomfortable (or overcrowding) areas, and split into three sections. The comfortable area means a lower load factor of transit lines in the area while the uncomfortable area means a higher load factor of transit lines.
**Step 2**. Many factors may affect passenger trip choice behavior. One is travel time that differs from one OD pair to the other. The comfort level in trip may also vary for different passengers and may be directly related to the fare. These factors can reflect the equity of the transit travel in urban transit system.

The investigations in the literature on these fare strategies have ascertained the superiority of differential fare strategy. Moreover, each strategy is compared with others in the literature in order to capture a more suitable fare structure.

The first contribution of the paper is to define a new area-based fare strategy in order to meet the request of equity at comfort level. Another contribution is the integration of the existing distance-based fare strategy and the new area-based fare strategy, aiming to obtain an appropriate combined fare structure. Such an area-based fare is similar to the structure of zonal fare and route-based fare. All these fare strategies are designated on the basis of zones or areas. These zones or areas can be divided in terms of load factor on the public transit corridor. However, these fare strategies are applied in different situation. For instance, zonal fare is usually adapted to the whole urban transit system, such as Dubai whose map is divided into 7 zones. Route-based fare is often present in some sections of an urban transit system. The area-based fare is proposed for the specified transit corridor.

The structure of this paper is as follows. Section 2 describes the scenario set up for this investigation, including representation of the bus-subway corridor and basic assumptions. Section 3 defines the components of the travel cost that influences passenger choice behavior. Section 4 presents model formulation and algorithms. Section 5 provides a set of numerical experiments to illustrate how we obtain the most suitable fare structure. Section 6 closes this paper with some concluding remarks.

## 2 Preliminaries

### Assumptions

**Fig 1:**
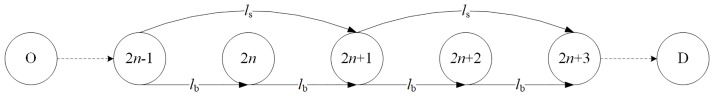
A sample bus-subway corridor.

The paper considers a corridor *G*(*N*, *L*_s_, *L*_b_) composed of a bus line and a subway line, as shown in Fig 1, where *N* is the set of nodes *n*, *L*_s_ is the set of segments of subway line between two successive stops and so is *L*_b_; *l*_s_ and *l*_b_ represent a subway and bus line segment respectively. It is assumed that each segment has the same length. Let *W* be the set of OD pairs, *w* represent an OD pair, and *M* be the set of paths for passengers. The bus line serves all OD pairs along the corridor, and the subway only serves part of the OD pairs.

To facilitate the presentation of the essential ideas and model formulation, some basic assumptions are made as follows:

*Transit line* The distance between two successive stations of subway line is twice the bus line. Each service has a constant frequency, and the average vehicle operating speed of each service is given and invariable.

*Passenger* All passengers are assumed to be homogeneous, i.e., they have an identical value of time. There are four alternative paths, namely, bus direct path, subway direct path and combined transfer paths that can be further divided into bus-subway and subway-bus transfer path. Passengers can complete their trips by all of these paths or part of them. With regard to transfer, passengers are also assumed never to use one mode for twice in trip, which means they can transfer once at most. Considering desire to the most favorable transfer, we assume that the alternative transfer path should be combined by most subway line segments and least bus line segments.

### Classification of OD pairs

According to Section 2.1, transit OD pairs in the bus-subway corridor are classified into three types in term of their alternative paths as shown in Table 1.

**Table 1:** Classification of OD pairs.

OD pairs	Transfer node	Origin	Destination	Direct path	Transfer path
		Subway line	Subway line		
Type one	Yes	Yes	Yes	Bus, Subway	Bus-Subway, Subway-Bus
	No	Yes	Yes	Bus, Subway	-
Type two	No	No	No	Bus	-
Type three	Yes	Yes	No	Bus	Subway-Bus
		No	Yes	Bus	Bus-Subway
	No	No	No	Bus	-

**Fig 2:**
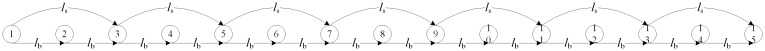
Bus and subway corridor.

*Type one* is the OD pairs whose origin and destination nodes are served by bus and subway lines, e.g. OD pair (2*n*-1, 2*n*+3) and (2*n*-1, 2*n*+1) in Fig 2. They can complete their trips by four alternative paths, namely, bus direct path, subway direct path and combined transfer path that can be further divided into bus-subway and subway-bus transfer path if there is at least one transfer node between the origin and destination nodes.

*Type two* is the OD pairs whose origin and destination nodes are served only by bus line, e.g. OD pair (2*n*, 2*n*+2) and (2*n*+1, 2*n*+2) in Fig 2. They have no alternative other than the bus direct path.

*Type three* is the OD pairs whose origin and destination nodes are served by bus line. Meanwhile, one of OD nodes is served by subway line, e.g. OD pair (2*n*-1, 2*n*+2) and (2*n*, 2*n*+3) in Fig 2. Except for the bus direct path, they have an alternative combined transfer path (bus-subway or subway-bus transfer path) if there is at least one transfer node between the origin and destination nodes.

The notations used throughout this paper are given in Table 2.

**Table 2:** Notations used throughout the paper.

*TW*	the walking time to enter and leave the station
*TTr*	the walking time to transfer between two service lines
*f*_*m*_	the frequency of transit service *m*
*L*_*m*_	the set of travel segments in transit line
*l*_*m*_	the unit travel segments in transit line
$d_{l_{m}}$	the length of each unit segment of transit service *m*
*V*_*m*_	the average vehicle operating speed of mode *m*
$\eta_{m}^{0}$	the baseline discomfort level of mode through segment when the vehicle is empty
$\eta_{m}^{1}$	the positive calibrated parameter of the in-vehicle discomfort function
$\nu_{l_{m}}$	he passenger flow in each unit segment *l*_*m*_
*k*_*m*_	the vehicle capacity of mode *m*
*A*	the set of area *a*(the comfortable area *a*1 and the uncomfortable area *a*2)
*S*	the set of section *s* (*s* = 1, 2,…, n) that can be obtained by splitting the corridor
$\omega_{s}^{L_{m}}$	a binary variable indicating the correlation between the traveling segments and the splitting section
$\mu_{a}^{s}$	a binary variable indicating the relevance between the splitting section *s* and the defining area
$\pi_{a}^{l_{m}}$	a binary variable indicating the correlation between the every transit segment and the defining area
$U_{M}^{w}$	the travel cost of travel paths *M*
*g*_*w*_	the total resultant passenger demand between OD pair *w*
$g_{w}^{0}$	the initial potential passenger demand between OD pair *w*
$h_{M}^{w}$	the passenger demand of alternative travel paths for OD pair *w*
Φ(**p**)	the total revenue of transit operators
**p**	the set of variable fares
*C*_*m*_	the cycle journey times for transit service *m*
*R*_*m*_	the number of vehicles for transit service *m*
*CT*_*m*_	the cycle journey times for transit service *m*
****$\varphi_{l_{m},M}$****	a binary variable that equals 1 if the segment of transit line *l*_*m*_ is part of path *M* and 0 if it is not

## 3 Travel cost modeling

It is assumed that there are four alternative paths: two direct paths (bus and subway) and two combined paths (bus-subway and subway-bus). Transit passengers make their path decision based on tradeoff between travel costs of their options. There are several basic components included in travel cost, e.g. walking time, waiting time, in-vehicle travel time, in-vehicle crowding discomfort, fare cost, which are defined as follows.

*Walking time* Considering the characteristics of subway stations, it takes passengers some walking time to enter and leave a station. The walking time at a bus station is not counted here because it is generally quite small. In addition, transit passengers also need some time to transfer between two service lines.

*Waiting time* The average waiting time a passenger spends in a station of transit mode *m* (b = bus, s = subway) can be calculated by

$$TW_{m}=\frac{\gamma_{m}}{f_{m}},m\in \left\{\text{b},\text{s}\right\}$$

where the parameter *γ*_*m*_ depends on the distribution of transit vehicle headways and passenger arrival time. Given the assumption of a uniform distribution of passenger arrivals and a constant transit vehicle headway, the value of *γ*_*m*_ adopted here is 0.5 [25].

*In-vehicle travel time* The average in-vehicle travel time in transit line segments *L*_*m*_, $T_{L_{m}}$, can be calculated as

$$T_{L_{m}}\text{=}\sum_{L_{m}}d_{l_{m}}/V_{m},\;m\in \left\{\text{b},\text{s}\right\},\;l_{m}\in L_{m}$$

*In-vehicle crowding discomfort* According to Spiess and Florian [10], the in-vehicle crowding discomfort cost is measured in terms of generalized time, and can be expressed in form of Bureau of Public Roads (BPR) type function with regard to the mean vehicle travel time, passenger volume, and vehicle capacity on the line. Considering the different automobile structure of two modes, an analogical uniform type function is applied to measure the cost of in-vehicle crowding discomfort in segment of transit line *l*_*m*_, and the discomfort can be expressed below:

$$UC_{l_{m}}=\left(\eta_{m}^{0}+\eta_{m}^{1}\left(\text{max}\left(0,\nu_{l_{m}}-f_{m}k_{m}\right)\right)\right)T_{l_{m}},m\in \left\{\text{b},\text{s}\right\}$$

where $T_{l_{m}}$ is the in-vehicle travel time in unit segment of transit line *l*_*m*_.

*Fare cost* The fare cost is associated with the fare strategy adopted. When traveling in segments of transit line *L*_*m*_, the fare cost $FC_{L_{m}}$ can be expressed in Table 3, in which, *p*_*m*_, $p_{l_{m}}$, $p_{m}^{a}$ and $p_{l_{m}}^{a}$ are the variable fares in the optimization problem under investigation, given the proposed fare strategies. Specifically, *p*_*m*_ is the fare of mode *m*, $p_{l_{m}}$ is the fare of unit segment in transit line *l*_*m*_, $p_{m}^{a}$ is the fare of mode *m* in area *a* and $p_{l_{m}}^{a}$ is the fare of unit transit segment *l*_*m*_ belonging to area *a*. Under area-based and combined fare strategies, the corridor is divided into two areas (comfortable area and uncomfortable area) and split into several sections. $\omega_{s}^{L_{m}}$ equals 1 if the traveling segments of transit line go through the section *s* and 0 if they don't. $\mu_{a}^{s}$ equals 1 if section *s* is part of area *a* and 0 if it is not. $\pi_{a}^{l_{m}}$ equals 1 if the transit segment *l*_*m*_ belongs to area *a* and 0 if it doesn't.

**Table 3:** Fare cost of service *m* under different strategy.

Fare strategy	Variable (fare)	Fare cost
Flat	*p*_*m*_ *m* = {b, s}	$FC_{L_{m}}=\begin{array}{cc} p_{m} & m=\left\{\text{b},s\right\} \end{array}$
Distance-based	$\begin{array}{cc} p_{l_{m}} & m=\left\{\text{b},s\right\} \end{array}$	$\begin{array}{cc} FC_{L_{m}}=\sum_{L_{m}}p_{l_{m}} & m=\left\{\text{b},s\right\} \end{array},\;l_{m}\in L_{m}$
Area-based	$\begin{array}{cc} p_{m}^{a} & m=\left\{\text{b},s\right\} \end{array},a\in A$	$\begin{array}{cc} FC_{L_{m}}=\sum_{s\in S}\sum_{a\in A}\omega_{s}^{L_{m}}\mu_{a}^{s}p_{m}^{a} & m=\left\{\text{b},s\right\} \end{array}$
Combined	$\begin{array}{cc} p_{l_{m}}^{a} & m=\left\{\text{b},s\right\} \end{array},a\in A$	$\begin{array}{cc} FC_{L_{m}}=\sum_{L_{m}}\sum_{a\in A}\pi_{a}^{l_{m}}p_{l_{m}}^{a} & m=\left\{\text{b},\text{s}\right\} \end{array},\;l_{m}\in L_{m}$

The travel cost of subway and bus direct paths (sd, bd) for each passenger between an OD pair *w* can be expressed in the following forms:

$$U_{M}^{w}=\{\begin{array}{ll} \alpha_{1}Tw+\alpha_{2}TW_{\text{s}}+\alpha_{3}T_{L_{\text{sd,s}}^{w}}+\alpha_{4}FC_{L_{\text{sd,s}}^{w}}+\alpha_{3}\sum_{l_{\text{sd,s}}^{w}\in L_{\text{sd,s}}^{w}}UC_{l_{\text{sd,s}}^{w}} & M\text{=}sd\\ \alpha_{2}TW_{\text{b}}+\alpha_{3}T_{L_{\text{bd,b}}^{w}}+\alpha_{4}FC_{L_{\text{bd,b}}^{w}}+\alpha_{3}\sum_{l_{\text{bd,b}}^{w}\in L_{\text{bd,b}}^{w}}UC_{l_{\text{bd,b}}^{w}} & M\text{=bd} \end{array},\forall w\in W$$

where the coefficients (*α*) are the reciprocal substitution factors between each cost component that is used to convert different quantities to the same unit. For the purpose of converting all different quantities to the equivalent in-vehicle travel time unit, the paper sets *α*_3_ equal 1.0. Then, *α*_1_ is the ratio of the value of walking time to the value of in-vehicle travel time; *α*_2_ is the ratio of the value of waiting time to the value of in-vehicle travel time, *α*_4_ is the reciprocal of the value of in-vehicle travel time. $L_{\text{sd,s}}^{w}$ and $L_{\text{bd,b}}^{w}$ are the set of segments in transit lines between OD pair *w* in two direct paths respectively. $l_{\text{sd,s}}^{w}$ and $l_{\text{bd,b}}^{w}$ are the unit segment in subway and bus line respectively. $T_{L_{\text{sd,s}}^{w}}$ and $T_{L_{\text{bd,b}}^{w}}$ are the in-vehicle travel time of traveling segments in subway and bus line respectively. $FC_{L_{\text{sd,s}}^{w}}$ and $FC_{L_{\text{bd,b}}^{w}}$ are the fare costs of subway and bus services when traveling in transit segments $L_{\text{sd,s}}^{w}$ and $L_{\text{bd,b}}^{w}$ in two direct paths respectively.

Based on the assumption, there are two transfer paths: bus-subway, bs, and subway-bus, sb. Their travel costs $U_{M}^{w}$, for each passenger between an OD pair *w*, can be expressed as

$$\begin{array}{l} U_{M}^{w}=\alpha_{1}\left(Tw+TTr\right)+\alpha_{2}\left(TW_{\text{s}}+TW_{\text{b}}\right)+TP+\\ \{\begin{array}{cc} \alpha_{3}\left(\sum_{l_{\text{bs,s}}^{w}\in L_{\text{bs,s}}^{w}}UC_{l_{\text{bs,s}}^{w}}+\sum_{l_{\text{bs,b}}^{w}\in L_{\text{bs,b}}^{w}}UC_{l_{\text{bs,b}}^{w}}\right)+\alpha_{3}\left(T_{L_{\text{bs,s}}^{w}}+T_{L_{\text{bs,b}}^{w}}\right)+\alpha_{4}\left(FC_{L_{\text{bs,s}}^{w}}+FC_{L_{\text{bs,b}}^{w}}\right) & M\text{=bs}\\ \alpha_{3}\left(\sum_{l_{\text{sb,s}}^{w}\in L_{\text{sb,s}}^{w}}UC_{l_{\text{sb,s}}^{w}}+\sum_{l_{\text{sb,b}}^{w}\in L_{\text{sb,b}}^{w}}UC_{l_{\text{sb,b}}^{w}}\right)+\alpha_{3}\left(T_{L_{\text{sb,s}}^{w}}+T_{L_{\text{sb,b}}^{w}}\right)+\alpha_{4}\left(FC_{L_{\text{sb,s}}^{w}}+FC_{L_{\text{sb,b}}^{w}}\right) & M\text{=sb} \end{array},\forall w\in W \end{array}$$

where the term *TP* is the transfer penalty which accounts for the resistance of passenger to change line other than the walking time [26]. $L_{\text{bs},s}^{w}$, $L_{\text{bs},\text{b}}^{w}$ and $L_{\text{sb},s}^{w}$, $L_{\text{sb},\text{b}}^{w}$ are the set of traveling segments in transit line s and b in each transfer path respectively.

## 4 Model formulation

The optimization of fare structure refers to the situation in which the whole bus-subway corridor is managed by an authority with an objective to maximize the total social welfare of the transit system. Under this condition, the transit operator determines the fare structure of each service. The passengers then make their choice according to the information determined. Furthermore, the authority’s decision must be influenced by the passenger's behavior, which illustrates that the authority should implement the reasonable decision about the fare structure in order to attract more demands. Thus, the models of optimization include two levels, of which the upper level aims to maximize the social welfare and the lower level is a variable-demand stochastic user equilibrium assignment model.

### Upper level

The objective of social welfare includes consumers surplus and producer profit. Although subsidy can be provided by the authority, it is not considered in the paper.

The total resultant passenger demand between OD pair *w* is assumed to be elastic and is specified as a negative exponential function with respect to the expected travel cost *E*_*w*_, i.e.,

$$g_{w}=g_{w}^{0}\text{exp}\left(-\beta E_{w}\right),\forall w\in W$$ (1)

where the parameter *β* (>0) reflects the demand sensitivity to the expected travel cost. According to the random utility theory, the expected travel cost can be measured by the following formula [27–29].

$$E_{w}=-\frac{1}{\theta }\text{ln}\left(\sum_{M}\text{exp}\left(-\theta U_{M}^{w}\text{+ln}PS_{M}^{w}\right)\right),M\in \left\{\text{bd},\text{sd},\text{bs},\text{sb}\right\}$$ (2)

where the parameter *θ* (>0) describes the variation of passenger perception on travel cost in the path choice decision. In accordance with the discrete choice theory, *θ*≥*β* must hold [28], $PS_{M}^{w}$ is the added term to the travel cost of alternative paths and will be described in next subsection.

The producer profit is the total revenue generated from passenger fares minus the total operating costs, and it can be expressed as

$$\Phi \left(p\right)=\sum_{w\in W}\left(h_{\text{bd}}^{w}FC_{L_{\text{bd,b}}^{w}}+h_{sd}^{w}FC_{L_{\text{sd,s}}^{w}}+h_{\text{bs}}^{w}\left(FC_{L_{\text{bs,s}}^{w}}+FC_{L_{\text{bs,b}}^{w}}\right)+h_{s\text{b}}^{w}\left(FC_{L_{\text{sb,s}}^{w}}+FC_{L_{\text{sb,b}}^{w}}\right)\right)-\left(R_{\text{s}}C_{\text{s}}+R_{\text{b}}C_{\text{b}}\right)$$ (3)

where $h_{\text{bd}}^{w}$, $h_{s\text{d}}^{w}$, $h_{\text{bs}}^{w}$ and $h_{\text{sb}}^{w}$ are, respectively, the passenger demand of bus direct path, subway direct path and two transfer paths for OD pair *w*. $FC_{L_{\text{bd,b}}^{w}}$, $FC_{L_{\text{sd,s}}^{w}}$, $FC_{L_{\text{bs,s}}^{w}}+FC_{L_{\text{bs,b}}^{w}}$ and $FC_{L_{\text{sb,s}}^{w}}+FC_{L_{\text{sb,b}}^{w}}$ are the corresponding fare costs. The number of vehicles for bus and subway service can be given by

$$R_{\text{b}}=f_{\text{b}}CT_{\text{b}},R_{\text{s}}=f_{\text{s}}CT_{\text{s}}$$ (4)

Consumer surplus, represented by $\sum_{w\in W}g_{w}/\beta$, is consumers' total benefits got from exchange, which can be, measured in time units following Williams [30] and Evans [31].

The objective function of the upper level model is the social welfare (*SW*) that is the sum of producer profit and consumers' surplus and represented mathematically in the following form:

$$\begin{array}{l} \text{max}SW\left(p\right)=\Phi \left(p\right)+\frac{\sum_{w\in W}g_{w}}{\alpha_{4}\beta }\\ \begin{array}{cc} s.t. & p\geq 0 \end{array} \end{array}$$ (5)

where **p** is the set of variable fares under the fare strategy described in Table 3. The remaining term, $\sum_{w\in W}g_{w}/\alpha_{4}\beta$, is the consumers’ surplus measured by time.

### Lower level

The lower level of the model reflects the passengers' response to the given fare structure. Considering the contradiction between the Independence from Irrelevant Alternative (IIA) property of multinomial logit formulation and the common segments among four alternative paths in the paper, what is applied here is a Path-Size Logit model [28], in which the probability $P_{M}^{w}$ that path *M* is chosen between OD pair *w* is defined by:

$$P_{M}^{w}=\frac{PS_{M}^{w}\text{exp}\left(-\theta U_{M}^{w}\right)}{\sum_{M}PS_{M}^{w}\text{exp}\left(-\theta U_{M}^{w}\right)},\forall w\in W,M\in \left\{\text{bd},\text{sd},\text{bs},\text{sb}\right\}$$ (6)

where $PS_{M}^{w}$ is the added term to the travel cost of alternative paths and it can be expressed by

$$PS_{M}^{w}=\sum_{l_{M,m}^{w}\in L_{M,m}^{w}}\left(\frac{t_{l_{M,m}^{w}}+u_{l_{M,m}^{w}}}{T_{M}^{w}+UC_{M}^{w}}\right)\frac{1}{\sum_{M}\delta_{M,l_{m}^{w}}},\forall w\in W,m\in \left\{\text{b},\text{s}\right\},M\in \left\{\text{bd},\text{sd},\text{bs},\text{sb}\right\}$$ (7)

where $L_{M,m}^{w}$ is the set of segments of transit line *m* in path *M* for OD pair *w*; $t_{l_{M,m}^{w}}$ and $u_{l_{M,m}^{w}}$ are respectively the in-vehicle travel time and in-vehicle crowding discomfort on each segment of transit line *m* in path *M* for OD pair *w*, $l_{m}^{w}$ is the unit segment of transit line *m* between OD pair *w*, and $\delta_{M,l_{m}^{w}}$ = 1 if $l_{m}^{w}$ is part of path *M*, and 0 otherwise. Besides, $T_{M}^{w}$ and $UC_{M}^{w}$ are respectively the total in-vehicle travel time and in-vehicle crowding discomfort on path *M* between OD pair *w*. These factors of travel cost are respectively the sum of travel time and crowding discomfort in each unit transit segment of related modes, which are saved as $t_{l_{M,m}^{w}}$ and $u_{l_{M,m}^{w}}$. If *M* is a direct path, $T_{M}^{w}$ and $UC_{M}^{w}$ are respectively the sum of the two factors in each unit segment of mode *b* or *s*. Otherwise, $T_{M}^{w}$ and $UC_{M}^{w}$ are respectively the sum of two factors in each unit segment of mode *b* and *s* that are involved in this transfer path.

Then, the passenger flow on path *M* can be computed by:

$$h_{M}^{w}=g_{w}P_{M}^{w}\begin{array}{cc} & \forall w\in W,M\in \left\{\text{bd},\text{sd},\text{bs},\text{sb}\right\} \end{array}$$ (8)

where *g*_*w*_ can be obtained by Eqs (1) and (2). Hence, the passenger flow on a segment of transit line *l*_*m*_ can be expressed by

$$v_{l_{m}}=\sum_{w\in W}\sum_{M}\varphi_{l_{m},M}h_{M}^{w}\begin{array}{cc} & \forall l_{m}\in L_{m},m\in \left\{\text{b},\text{s}\right\},M\in \left\{\text{bd},\text{sd},\text{bs},\text{sb}\right\} \end{array}$$ (9)

The lower level of this model is composed by Eqs (6), (8), (9) plus the following nonnegativity constraints:

$$h_{M}^{w}\geq 0,\forall w\in W,M\in \left\{\text{b},\text{s},\text{c}1,\text{c}2\right\}or\left\{\text{b},\text{s}\right\}$$ (10)

$$g_{w}\geq 0,\forall w\in W$$ (11)

This is a route choice model represented by a set of constraints, which can be reformatted as an optimization model whose first-order necessary conditions are the set of constraints.

## 5 Solution algorithm

Considering the complexity of the nonlinear optimization problem, a genetic algorithm (GA) with double-point crossover is administrated to solve the bi-level program. Meanwhile, the method of successive averages (MSA) [27] is adopted to solve the lower-level model.

The GA algorithm applied is demonstrated as follows:

**Step 3**. : Initialization. Choose the values of relevant parameters in GA, such as the population size, the maximum generation *NG*, the probability of performing crossover and mutation; select the range of variables *p*_*m*_, *λ*_*m*_; save *n* = 1 for the loop time.
**Step 4**. : Computation. Calculate the travel cost of each path, the selective probability and the passenger flow on path for each chromosome; count the objective function of each individual chromosome *SW*(*i*); identify the maximum and minimum objective function of population saving as *SW*_*max*_ and *SW*_*min*_; define the feature of each individual chromosome as *SW*(*i*)-*SW*_*min*_.
**Step 5**. : Operation. Perform selection, reproduction, crossover and mutation procedures.
**Step 6**. : Verification. Terminate the operation when the loop time reaches the maximum generation, and output the data *SW*_*max*_ in lasted population and other variables needed. Otherwise, repeat step2.

The MSA algorithm applied can be summarized as follows:

**step1** : Initialization. Perform a stochastic network loading based on a set of initial travel cost *U*. Then generate a set of passenger flow on four paths *HA*. Set *n* = 1.
**step2** : Update. Set *U*^(*n*)^ *= U*(*HA*^(*n*)^).
**step3** : Direction finding. Perform a stochastic network loading procedure based on the current set of path travel costs *U*^*n*^, then yield an auxiliary passenger flow pattern *HB*^(*n*)^.
**step4** : Move. Set the new flow pattern as *HA*^(*n*+1)^ *= HA*^(*n*)^+(1/*n*)(*HB*^(*n*)^-*HA*^(*n*)^)
**step5** : Convergence criterion. If convergence is attained, stop. Otherwise, set *n* = *n*+1 and go to step 2.

## 6 Numerical experiments and analysis

To facilitate the presentation of the essential ideas and contributions of this paper, we apply the models proposed and the solution algorithms to an example bus-subway corridor. The numerical experiments is used to not only illustrate the advantage of differential fare strategy comparing with flat fare strategy, but also ascertain the superiority of combined fare strategy in meeting request of equity.

### Data input

The example corridor, as is shown in Fig 2, consists of 15 nodes: one bus line that serves all nodes, one subway line that serves part of nodes, and a unidirectional passenger demand given in Table 4. The distances between two successive stops of bus and subway line are 0.6km and 1.2km respectively. In other words, the length of *l*_s_ is the double that of *l*_b_. Each service line has its own stations. The walking time in subway station *Tw* is 0.06h. There is a walking distance between different service stations on the same corridor node, which is uniform in the whole corridor and measured by transfer walking time *TTr* = 0.1h. Other parameter values for the numerical experiments are given in Table 5. The values used for capacities, operating costs, average operating speeds and frequencies of vehicle for two services are displayed in Table 6.

**Table 4:** Potential demand in transit corridor (pax/h).

**O**	**1**	**2**	**3**	**4**	**5**	**6**	**7**	**8**	**9**	**10**	**11**	**12**	**13**	**14**	**15**
**D**															
**1**	0	210	1786	118	1448	112	1474	99	1465	83	1497	82	1601	66	1575
**2**	-	0	143	227	227	182	182	252	252	174	174	121	121	126	126
**3**	-	-	0	261	1799	129	1955	124	3074	91	2522	88	2159	12	3163
**4**	-	-	-	0	205	148	148	141	141	141	140	113	112	91	91
**5**	-	-	-	-	0	115	1390	134	1579	107	1272	103	1427	96	334
**6**	-	-	-	-	-	0	94	104	104	121	120	111	111	111	111
**7**	-	-	-	-	-	-	0	108	1393	116	1546	117	1505	136	1564
**8**	-	-	-	-	-	-	-	0	100	89	90	117	117	114	114
**9**	-	-	-	-	-	-	-	-	0	102	1183	115	1451	109	1638
**10**	-	-	-	-	-	-	-	-	-	0	178	155	126	143	148
**11**	-	-	-	-	-	-	-	-	-	-	0	136	1147	111	1414
**12**	-	-	-	-	-	-	-	-	-	-	-	0	130	179	176
**13**	-	-	-	-	-	-	-	-	-	-	-	-	0	181	1604
**14**	-	-	-	-	-	-	-	-	-	-	-	-	-	0	90

**Table 5:** Values of parameter.

Parameter	*α*_1_	*α*_2_	*α*_3_	*α*_4_	*α*_5_	*γ*_b_	*γ*_s_	*β*	*θ*	$\eta_{\text{b}}^{0}$	$\eta_{\text{b}}^{1}$	$\eta_{\text{s}}^{0}$	$\eta_{\text{s}}^{1}$
Value	1.2	2.0	1.0	0.125	0.8	0.5	0.5	0.6	3.5	0.5	0.1	0.1	0.02

**Table 6:** Characteristics of bus and subway services.

Mode	Bus	Subway
Vehicle capacity *k*_*m*_ (pax/veh)	120	600
Operating cost *C*_*m*_ (¥/veh-h)	80	900
Average operating speed *V*_*m*_ (km/h)	10	30
Frequency *f*_*m*_ (veh /h)	60	20

### Numerical analysis

This section presents numerical experiments to illustrate how to use the formulated model to determine the most suitable fare structure and carry out a series of numerical analysis.

**Step 1.** *Flat fare strategy*

**Fig 3:**
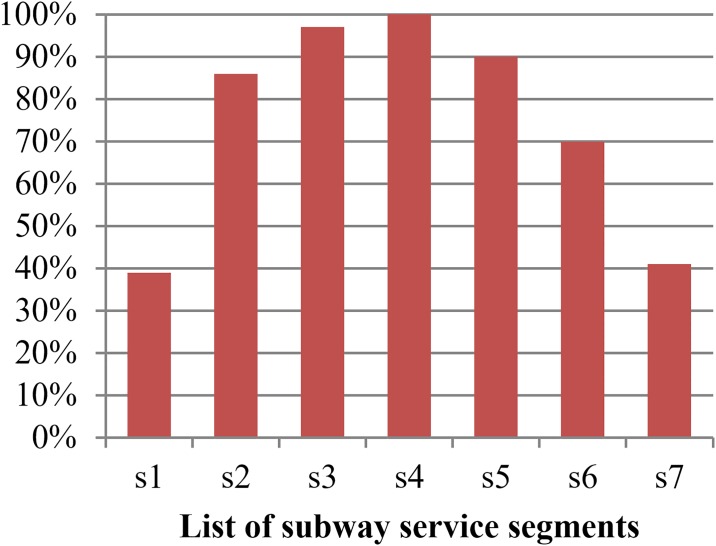
Load factor in segment of subway line.

**Fig 4:**
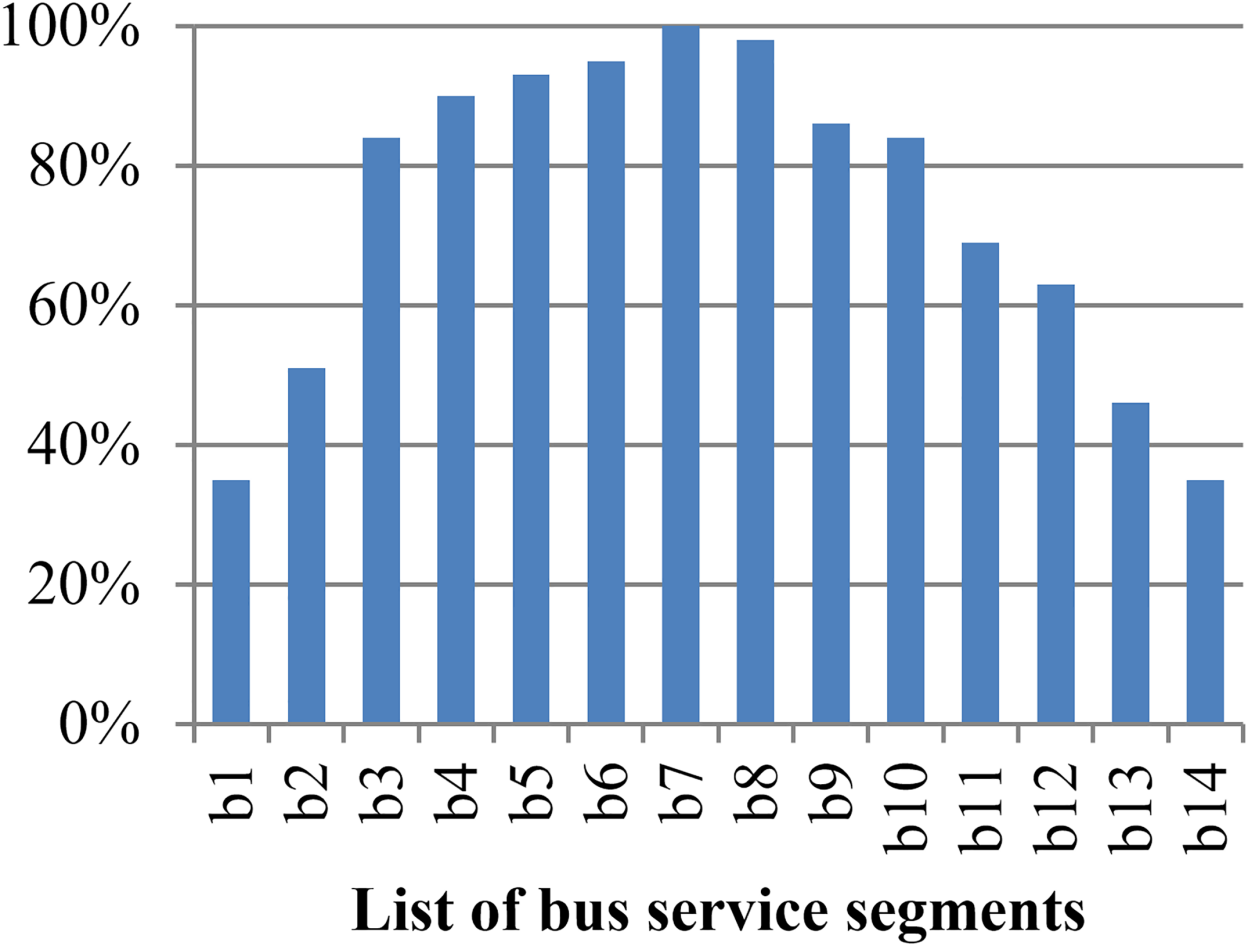
Load factor in segment of bus line.

We first implement the flat fare strategy in the bus and subway corridor with an aim to determine a reference level of social welfare and ascertain the general distribution of passenger flow. The results under the flat fare strategy are displayed in Table 7. With the optimal fares in Table 7, the load factor of subway and bus lines that reflect the passenger flows in segments of transit lines are shown in Figs 3 and 4, respectively.

**Table 7:** Results under flat fare strategy.

Strategy	Fare *p*_*m*_ (¥)		Passenger demand(pax/h)	*SW*(¥/h)
	Bus	Subway		
Flat	3.3	3.3	35999	597509

**Fig 5:**
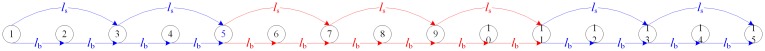
Distribution of different areas along a corridor.

The number of OD pairs served by a transit segment increases from both ends to the middle of the transit corridor. For example, the segment of bus transit line b1 serves total 14 OD pairs (1,2), (1,3),…, (1,15); b14 serves another total 14 OD pairs (1,15), (2,15),…, (14,15); b7 serves total 48 OD pairs (1,8), (1,9),…, (1,15), (2,8), (2,9),…, (2,15), (3,8), (3,9),…, (3,15) and so on. As shown in the two figures, the load factors of the two services have the highest level in the middle segments of transit lines, and the lowest level at both ends of the corridor. The sharp increase in the load factor for the front end of transit lines can reflect the ever-increasing passenger demand. However, restrained by the capacity of transit vehicles and affected by in-vehicle crowding, the growth of load factor is slowed down in the segments closing to middle of corridor and reaches the peak in segments of middle of transit lines. The level of load factor declines gradually in segments from the middle to the back end of transit lines in corridor. In terms of the load factor in transit lines, the corridor can be divided into two areas: the comfortable area, including both ends of the corridor and the uncomfortable area, including the segments of transit lines near the middle of corridor. As show in Fig 5, the comfortable areas include the range from node 1 to node 5 and from node 11 to node 15, in which the segments of transit lines are highlighted in blue, and the other area covering the range from node 5 to node 11 are red.

**Step 2.** *Differential fare strategy*

Three differential fare strategies, defined in introduction section and proposed in this section, aimed to obtain a suitable fare structure for the corridor are an existing distance-based fare strategy, a new area-based fare strategy and a combination of these two. The optimal results under differential fare strategies are displayed in Table 8.

**Table 8:** Results under differential strategy.

Fare strategy	Transit service	Variable (fares)	Results (¥)	Passenger demand(pax/h)	*SW*(¥/h)
Distance-based	Bus	$p_{l_{\text{b}}}$	0.6	37202	606505
	Subway	$p_{l_{\text{s}}}$	1.3		
Area-based	Bus	$\left\{p_{\text{b}}^{a1},p_{\text{b}}^{a2}\right\}$	{1.4, 2.3}	36479	604888
	Subway	$\left\{p_{\text{s}}^{a1},p_{\text{s}}^{a2}\right\}$	{1.6, 2.7}		
Combined	Bus	$\left\{p_{l_{\text{b}}}^{a1},p_{l_{\text{b}}}^{a2}\right\}$	{0.5, 0.5}	38638	611449
	Subway	$\left\{p_{l_{\text{s}}}^{a1},p_{l_{\text{s}}}^{a2}\right\}$	{0.8, 1.3}		

First, we check the value of objective function and the result passenger demand under differential fare strategy. As shown in Table 8, both the result social welfare and passenger demand under three differential fare strategies are higher than the results under flat fare strategy in Table 7. Hence it is better to consider the equity in trip when adopting a reasonable fare strategy. The highest level of result social welfare and passenger demand under the combined fare strategy illustrate that the more factors a differential fare strategy involves, the more suitable fare structure the strategy has. The optimal fare structure is the most suitable fare structure among four fare strategies for the bus and subway corridor.

Two areas of bus line have the same optimal fare under combined strategy. These optimal fares are so close to the optimal fare under distance-based fare strategy that we can conclude that the distance-based fare strategy is also fit for the bus service completely. Moreover, the distance-based fare strategy is easier to operate in actual bus service system.

**Fig 6:**
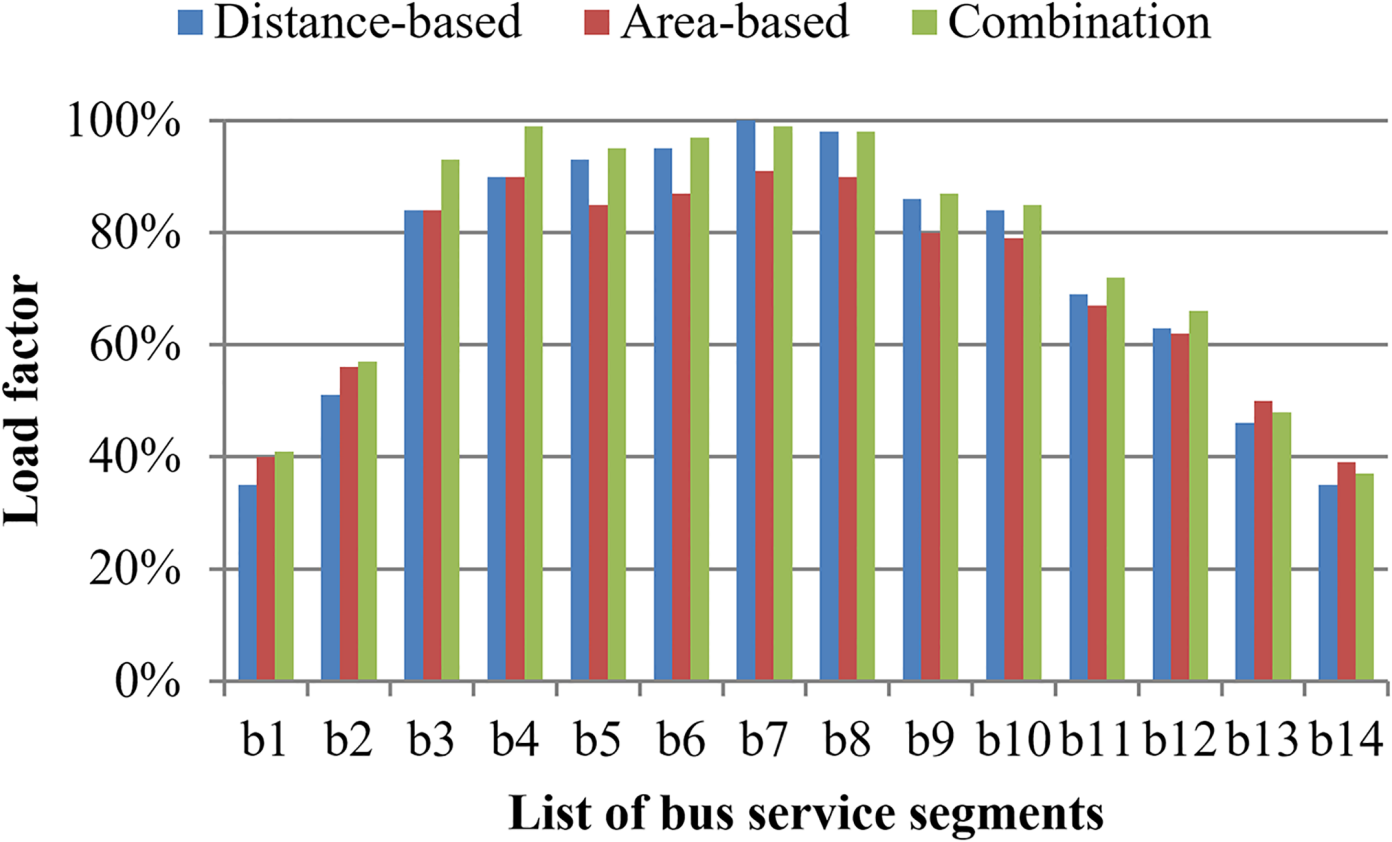
Load factor in segment of bus line under differential fare strategies.

The implementation effect of three differential fare strategies can be reflected by the load factor in segments of transit lines. Taking the bus line for example, the load factors in segments are exhibited in Fig 6.

As shown in Fig 6, under the distance-based fare strategy, the level of load factor keeps the rising tendency in the front half part of a bus line and the declining tendency in the back part of a bus line. For the other two differential fare strategies that involve the comfort factor, the level of load factor falls back in the segment accessing the uncomfortable area. For example, the load factors in segment b4 are 99% and 93% respectively for the area-based and combined fare strategies. The value falls down to 93% for the area-based fare strategy, and holds the same level for the combined fare strategy. The changing trend of the load factor reverses in the segment accessing the uncomfortable area under the area-based fare strategy and is fixed under the combined fare strategy. Then, the load factor increases again from bus segment b5 to b7, e.g. from 95% to 99% for the area-based fare strategy and from 93% to 100% for the combined fare strategy. The comparison among the three strategies illustrates that the fare strategies involving the comfort level factor play an important role in regulating the load factor in uncomfortable (or overcrowded) area. We can also see that the level of load factor under the combined fare strategy is the highest in major segments of bus line. This comparison also indicates the superiority of the combined fare strategy. Moreover, all OD pairs are influenced by travel time while some parts of OD pairs are impacted by the crowding discomfort along a corridor. Therefore, all OD pairs benefit from the distance-based fare with respect to the equity of travel time; part of the OD pairs benefit from the area-based fare in terms of the equity of comfort level. Most benefits can be captured by applying the combined fare strategy, because all OD pairs can be benefited from taking the two factors related to equity into account.

**Fig 7:**
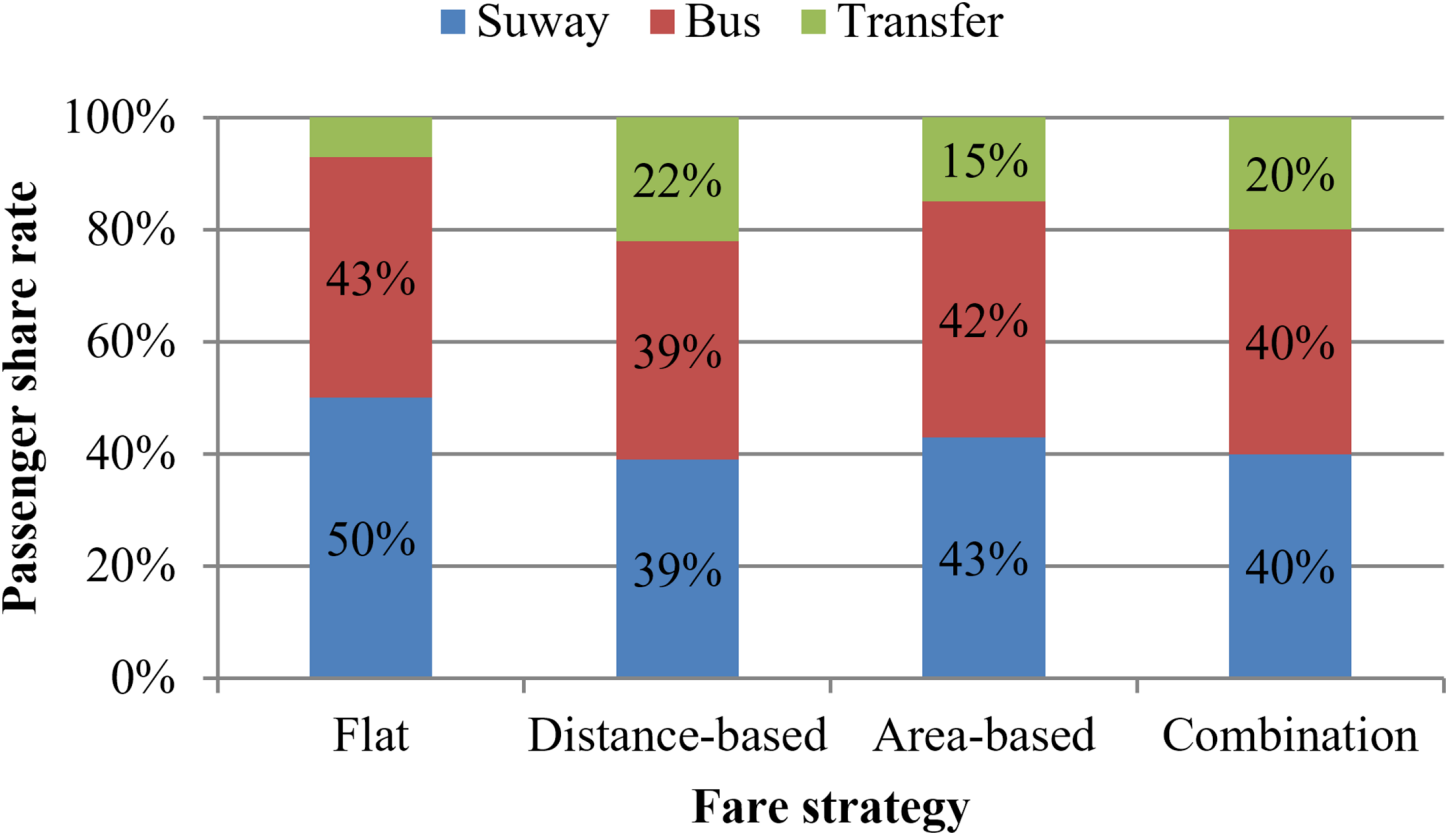
Passenger shares for three paths.

Then, we analyze the passenger behavior under four fare strategies proposed here. As showed in Fig 7, the passenger share rates of three paths, including bus direct, subway direct and transfer path.

For the flat fare strategy, the optimal fares of bus and subway are similar while the travel time of subway is much less than the bus in the same travel distance. It leads to a higher passenger share rate of subway path and lower passenger share rate of bus path. The passenger share rate of transfer path is the lowest in four fare strategies because fare cost is saved barely compared with two direct paths.

For the distance-based fare strategy, the optimal fare of subway service is twice those of bus service. The inferior of fare can offset the superiority of travel time in part comparing with bus service. Thus, the passenger share rate of subway direct path decreases significantly referring to the share rate under flat fare strategy. The fare costs of transfer paths decline by a great degree compared with the transfer cost under flat strategy, which results in a higher passenger share rate of transfer path.

For the area-based fare strategy, the passenger share rates of three travel paths are similar with flat fare strategy. The corridor under flat fare strategy can be considered as one area. With the optimal fares in area-based fare structure, the fare costs of two direct paths are more than that of the flat fare strategy for the trips through more than one split section. Because of more rise in fare cost for subway direct path, its passenger share rate declines to a certain extent. The passenger shares decreased switch to other paths. Moreover, the fare costs of transfer paths are much lower than that of the flat fare strategy, which leads to a significant rise in passenger share of transfer and a slight rise in bus direct paths.

For the combined fare strategy composed by distance-based and area-based fare strategies, the passenger share among the three paths are similar to the situation under the distance-based fare strategy. The optimal subway fare in comfortable area has a greater degree of decline while the bus fare in this area is lower than that under the distance-based fare strategy. Thus, the passenger share of subway direct paths is higher than the distance-based fare structure. As shown in Fig 7, although the passenger share of transfer paths under the area-based fare strategy is less than that under the distance-based fare structure, the actual passenger demand of transfer paths under the combined fare strategy increases 319 pax/h comparing with that under the distance-based fare strategy. The decrease in passenger share of transfer paths is ascribed to the responsive rise of the total passenger demand (about 1380pax/h).

It can be seen in Fig 7 that the passenger share rate of transfer has the highest degree in distance-based and combined fare structure. Meanwhile, the result social welfare and passenger demand have higher levels under two strategies.

**Fig 8:**
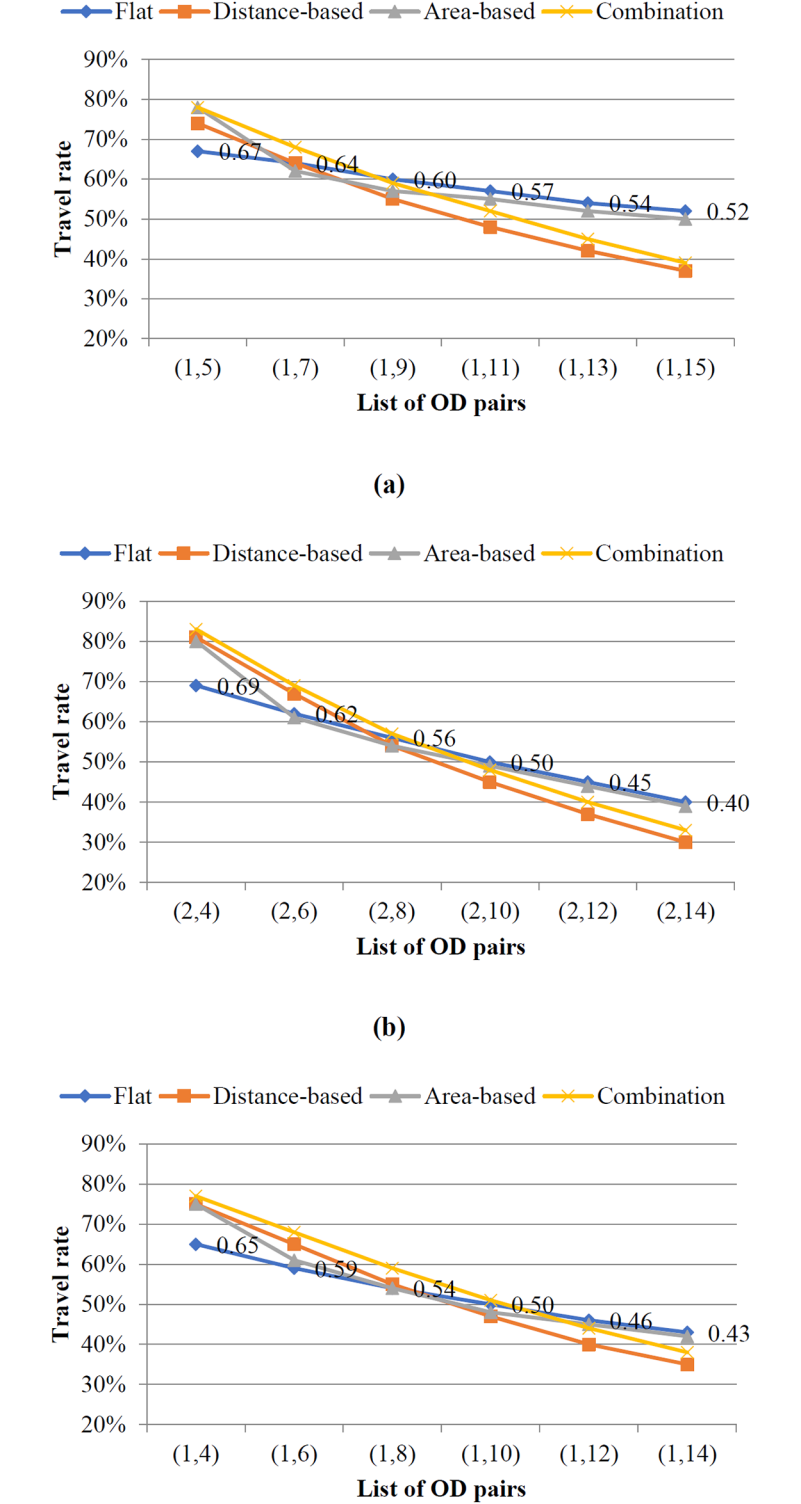
Travel rates for different types of OD pairs. (a) Type One OD pairs. (b) Type Two OD pairs. (c) Type Three OD pairs.

We also observe more details of passenger behavior referring to the classification of OD pairs defined in Table 1. Three groups of OD pairs belong to the relevant type are taken for example. The travel rates of three types OD pairs are showed in (Fig 8a, 8b and 8c). As shown in Fig 8, as the travel distance increases, the travel rates of three types OD pairs decline. For the shortest travel distance, the fare cost with flat fare is the highest, so the travel rate with flat fare is the lowest compared in all fare strategies. For the longest travel distance, the fare costs with distance-based and combined fare strategies are the higher, so the travel rates under two fare strategies are lower than other fare strategies. The distance-based and combined fare strategies have the similar decline rates which are higher than other fare strategies because of the increasing fare costs. The area-based fare strategy has the similar decline degrees with the flat fare strategy for longer travel distance, like the rate change among OD pair (1,11), (1,13) and (1,15). Furthermore, under area-based fare strategy, the fluctuating decline extents in the OD pairs that enter into and pass through the uncomfortable area, like (1,7) and (1,11), (2,6) and (2,10), (1,6) and (1,10), reflect the significant change in fare cost. Although this change can be present in combined fare strategy, the lower fares of unit segment weaken the decline extent. The change of travel rate in combined fare structure shows the similar characteristics with the distance-based fare structure. Therefore, the combined fare strategy as well as the distance-based fare strategy can meet the request of equity in travel distance.

Considering the classification of OD pairs, both type one and three OD pairs have at least two types of paths to choose while type two OD pairs can only travel by bus direct paths. That is why the difference of travel rates between the OD pair with the longest travel distance and the shortest travel distance is the largest for type two OD pairs. It can be verified by the change of travel rates in flat fare strategy as an example. As we can see, the difference of travel rate is 12% between type three OD pair (1,4) and (1,14), 15% between type one OD pair (1,5) and (1,15) and 29% between type two OD pair (2,4) and (2,14).

**Step 3.** *Sensitivity Analysis of Passenger Demand*

If more than one transit mode is involved in a public transit system, there exist common OD pairs served by all transit modes, such as the *Type one* set of OD pairs. In this subsection, only passengers between the *Type one* OD pairs are treated as the potential demand and only the distance-based and combined fare strategies are considered due to the superiority of these strategies. The optimal results under differential fare strategies are displayed in Table 9.

**Table 9:** Results under differential strategy (*Type one* OD pairs).

Fare strategy	Transit service	Variable (fares)	Results (¥)	Passenger Demand (pax/h)	*SW*(¥/h)
Distance-based	Bus	$p_{l_{\text{b}}}$	0.1	35351	511889
	Subway	$p_{l_{\text{s}}}$	0.7		
Combined	Bus	$\left\{p_{l_{\text{b}}}^{a1},p_{l_{\text{b}}}^{a2}\right\}$	{0.14, 0.14}	38638	514624
	Subway	$\left\{p_{l_{\text{s}}}^{a1},p_{l_{\text{s}}}^{a2}\right\}$	{0.3, 0.8}		

Comparing with Step 2 shows that the total demand decreased and that the fare for each service in Table 9 is lower than that in Table 8 under the given fare strategies. The eliminated two types of OD pairs relate the bus line directly because the passengers between the type two OD pairs must complete their trips by direct bus paths and those between the type three OD pairs can't access direct subway paths. Thus, the gaps in the fare between subway and bus services are narrowed down. Moreover, the gaps between Tables 7 and 8 are the same for the fares in each area of *a*1 and *a2* under the combined fare strategy, i.e. the bus fares for the two areas changed respectively from 0.5¥ and 0.5¥ to 0.14¥ and 0.14¥ (both decreased by 0.36¥), and the subway fares for the two areas fell respectively from 0.8¥ and 1.3¥ down to 0.3¥ and 0.8¥ (both decreased by 0.5¥). These changes in the fare suggest that combined fare strategy be adaptable for different types of OD pairs or different passenger demand levels. In addition, the combined fare strategy can result in the better social welfare and passenger demand than the other one. The results in Table 9 prove the superiority of the combined fare strategy again.

The transit corridor defined here is the same as one in [32], in which more sensitive analysis can be found, including the influence of transit vehicle capacity and passenger demand level.

## 7 Concluding remarks

A bi-level program has been formulated in this paper to optimize the desirable fare structure along a bus-subway corridor with an objective to maximize the social welfare. For the purpose, a flat fare strategy is applied to gather information on passenger flow in transit lines and confirm a bottom line for the social welfare. Then, three differential fare strategies are proposed to address the concern with equity in trip and compared to determine the most suitable fare structure. The distance-based fare strategy considers the equity of travel distance for passengers, the area-based fare strategy considers the comfort level in trip, and the combined fare strategy relates both distance and comfort factors to the equity issue in trip.

In order to find the desirable fare structure, we examine the optimal fare structure under different fare strategies by such measures as social welfare and passenger demand. Numerical experiments have been designed to carry out further analysis and here is a list of main findings:

1. All three differential fare strategies play a better role in promoting social welfare and have a more suitable fare structure than the flat one.
2. The combined fare strategy has led to the highest social welfare and resultant passenger demand, which implies that the more equity factors a differential fare strategy is involved with the more suitable fare structure the strategy has.
3. The similar result fare structure of bus services between distance-based and combined fare strategies suggests that the distance-based fare strategy is also fit for bus service completely.

The major challenge in this investigation is how to split the reasonable range of two areas. We split the corridor into three sections, i.e. the front, middle and back end. The fare of each section may be different but the fares for both end sections are kept the same and designated as one variable in this paper. The discussion on the passenger behavior for each OD pair type is not part of this paper.

Public transit has been widely discussed and used around the world to address traffic congestion or travel demand management. In addition to identification or optimization of desirable fare strategies, giving the priority of green traffic light time or the right of the road to public transit is also a hot topic in the current literature and practice of traffic and transportation engineering. When we discuss public transit service pricing, traffic congestion pricing is another topic on pricing [33–34], which both aim to maximize the social welfare but mainly target respectively at public and private transportation.
